# Viscoelastic Polyurethane Foams for Use as Auxiliary Materials in Orthopedics

**DOI:** 10.3390/ma15010133

**Published:** 2021-12-24

**Authors:** Dominik Grzęda, Grzegorz Węgrzyk, Milena Leszczyńska, Leonard Szczepkowski, Michał Gloc, Joanna Ryszkowska

**Affiliations:** Faculty of Materials Science and Engineering, Warsaw University of Technology, Wołoska 141, 02-507 Warsaw, Poland; dominik.grzeda.stud@pw.edu.pl (D.G.); grzegorz.wegrzyk.dokt@pw.edu.pl (G.W.); leonardosz@interia.pl (L.S.); michal.gloc@pw.edu.pl (M.G.)

**Keywords:** viscoelastic polyurethane foams, limb prosthesis, prosthesis liner

## Abstract

One of the essential factors in prostheses is their fitting. To assemble a prosthesis with the residual limb, so-called liners are used. Liners used currently are criticized by users for their lack of comfort, causing excessive sweating and skin irritation. The objective of the work was to develop viscoelastic polyurethane foams for use in limb prostheses. As part of the work, foams were produced with different isocyanate indexes (0.6–0.9) and water content (1, 2 and 3 php). The produced foams were characterized by scanning electron microscopy, computer microtomography, infrared spectroscopy, thermogravimetry and differential scanning calorimetry. Measurements also included apparent density, recovery time, rebound elasticity, permanent deformation, compressive stress value and sweat absorption. The results were discussed in the context of modifying the foam recipe. The performance properties of the foams, such as recovery time, hardness, resilience and sweat absorption, indicate that foams that will be suitable for prosthetic applications are foams with a water content of 2 php produced with an isocyanate index of 0.8 and 0.9.

## 1. Introduction

**Figure 1:**
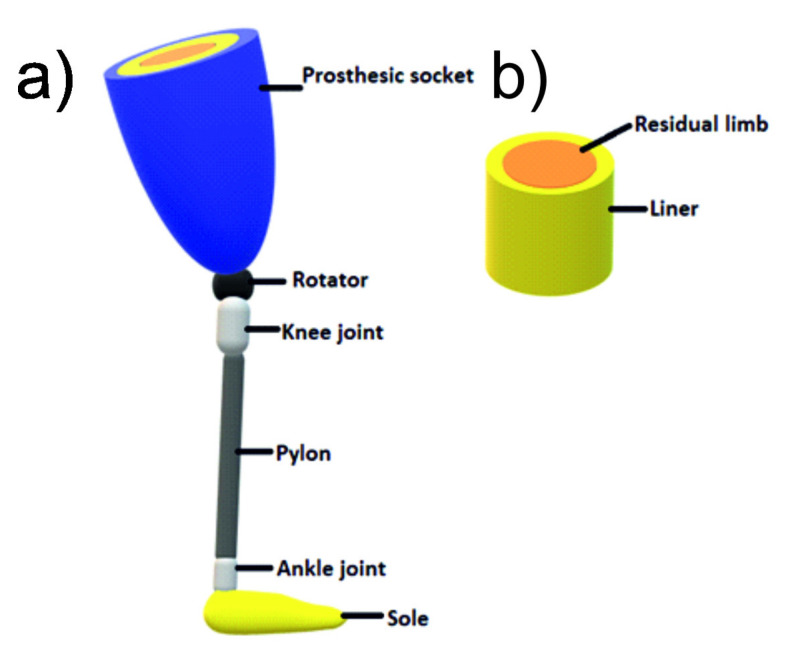
Simplified prosthesis structure: structure (**a**) and stump with liner (**b**) [3].

In 2017, 57.7 million people lived after limb amputation due to traumatic causes worldwide [1]. In the USA, there are about 2 million cases, of which 45% are traumatic, and the majority, 54%, are caused by vascular diseases [2]. A prosthesis improves the comfort of these people’s lives. Description of the structure of an exemplary leg prosthesis is shown in Figure 1.

An essential role for prosthesis users is a so-called liner located in the socket of the prosthesis [3]. It improves the comfort of wearing the prosthesis, reduces the stress transmitted by the stump, and makes a better prosthesis fit. In commonly used liner materials such as silicone and elastomers [4], amputees complain about thermal discomfort and increased perspiration due to their poor thermal conductivity, thus insulating the residual limb. The design in which the socket, liner and limb residue are in complete contact ensures the correct distribution of pressure between the limb and the prosthesis [5]. However, limb residual tends to change its volume and size, especially during activities, focusing the prosthesis pressure on particular stump areas, which causes pain and injuries. Due to long-time contact with the skin, previous liners often cause skin irritation and lead to skin diseases and damages. Such changes occur mainly in the vicinity of bone protrusions. In such places, the network of vessels is compressed, limiting the supply of oxygen and essential nutrients, consequently leading to ischemia of soft tissues and ultimately to their disintegration [6,7].

According to the review of prosthesis satisfaction [8], polyethylene foam liners were the most approachable during donning and doffing [9]. Analyses of satisfaction of amputees with the prostheses used indicate that it is lower than desired by the medical services industry and the clinical community [10].

Therefore, the work proposes using viscoelastic polyurethane foams, also known as shape memory foam, to produce an intermediate layer between the liner and the limb. The usefulness of these foams has been verified, among others, for the production of anti-bedsore mattresses and shoe inserts, pressure bandages [6,11].

Study of Bai et al. [12] has shown that viscoelastic polyurethane foam used in mattresses can postpone the occurrence of pressure injuries by 4.2 days on average compared to nonpressure redistributing foam mattress. It is likely that a similar phenomenon can be achieved with a viscoelastic foam layer in a prosthesis.

The properties of these foams significantly depend on the selection of the ingredients and their proportions; therefore, to verify the idea of producing the discussed elements of prostheses from these innovative foams, it is necessary to study their recipes. Typically viscoelastic foams are produced using an isocyanate index (INCO) = 0.6–1.05, using water as the chemical blowing agent. Their processing properties depend on the viscosity and reactivity of the polyols, the isocyanate index and the blowing agent content. The strength properties of foams depend on the molecular weight, functionality, and structure of polyols and their proportions and the content of isocyanate and water (H_2_O) [9,10].

The study assumes that the same polyol component will be used to produce foams, and the variables will be INCO and H_2_O content.

## 2. Materials and Methods

### 2.1. Materials

As part of the work, a series of 12 viscoelastic foams with a water content of 1.0, 2.0 and 3.0 php (parts according to one hundred parts of polyol, *wt*/*wt*) and isocyanate indexes (INCO) = 0.6; 0.7; 0.8 and 0.9. Polyol masterbatches (component A) were prepared by mixing the substrates: polyols, catalysts, surfactant and water according to a formula reserved by the company Fampur (Bydgoszcz, Poland). Characteristics of the prepared premixes are given in Table 1.

The isocyanate component (B) was polymeric methylene diphenyl diisocyanate (pMDI) (BorsodChem, Kazincbarcika, Hungary), commercially traded as Ongronat 4040 TR, containing 32.6% of free isocyanate groups at a calculated equivalent value of R_NCO_ = 128.8. The synthesis procedure was described in the Section S1 of Supplementary Data.

The markings, the foam components ratio, INCO index, and water content are given in Table 2.

### 2.2. Synthesis Parameters

An electronic stopwatch was used with an accuracy of 1 s to determine the cream time (reaction initiation time), rise time (to maximum foam height), and gel time (to the time when the mixture viscosity is sufficient for pulling a filament from the polymer using a rod).

### 2.3. Apparent Density

The apparent density was calculated by measuring the weight and volume of the sample according to EN ISO 845 [13]. The weight of the samples was determined with an accuracy of ±0.1 mg using WPA 180/C/1 analytical balance (Radwag, Radom Poland), and 50 mm × 50 mm × 50 mm cubes cut from the samples were measured with an accuracy of ±0.1 mm.

### 2.4. Gel Fraction

The extraction experiments in *N*,*N*-dimethylformamide (DMF) solvent (Chempur, Piekary Śląskie, Poland) were conducted to evaluate the crosslinking level (gel fraction). Three samples of each studied foam (approx. 200 mg) were prepared, weighted (m_0_), and immersed in DMF for 24 h. Afterwards, the samples were taken out from the DMF and washed twice by immersing the sample in acetone (Chempur, Piekary Śląskie, Poland) for 5 min. Excess solvent was blotted on cellulose sheets. The samples were dried at 25 °C under vacuum until the constant weight was achieved. The weight of the dry samples (m) was determined, and the gel fraction was calculated as the ratio between the dry mass of the sample after extraction (m) and the dry mass of the sample before extraction (m_0_).

### 2.5. Fourier Transform Infrared Spectroscopy (FTIR)

The chemical composition of the foams was analyzed using absorption spectra obtained with a Nicolet 6700 spectrophotometer (Thermo Electron Corporation, Waltham, MA, USA) equipped with an attenuated total reflection (ATR) module. Each sample was scanned 64 times in the wavelength range of 4000–400 cm^−1^. The results were analyzed with Omnic Spectra 8.2.0 software (Thermo Fisher Scientific Inc., Waltham, MA, USA). For each RL foam, a representation of three spectra was taken.

### 2.6. Scanning Electron Microscopy (SEM)

Samples were observed using a Hitachi TM3000 (Hitachi High-technology Corporation, Toranomon Minato-Ku, Japan). Before observation, the samples were sprayed with a palladium-gold layer. Imaging was performed with secondary electrons at an acceleration voltage of 5 kV. Porosity was assessed in pore size, shape, and spatial distribution based on several images taken at ×40 magnification. SEM images were used to calculate the mean equivalent diameter and anisotropy index of pores (N ≥ 150 for each foam variant). The anisotropy index was calculated as the ratio of the cell height to width.

### 2.7. Microtomography (µCT)

The cell size distribution and porosity of the foams were determined by Xradia 400CT tomograph device (Zeiss, Jena, Germany) equipped for computer software suited for image reconstruction. Cubic samples with dimensions of 2 cm × 2 cm × 2 cm were tested. The X-ray beam was generated using 40 kV accelerating voltage and power of 10 W. In total, 1261 frames were taken, which constituted to 180° rotation of the sample. Each frame was collected in 3 s. The #LE1 filter was used. Total scanning time for each sample was about 4 h, including reference frames of the air every 8 min. The detector position was selected to enhance the phase contrast. The image reconstruction was performed using the integrated X-Radia reconstructor software (Zeiss, Jena, Germany). Depending on the sample, the center shift parameter was introduced as well as the beam hardening parameters. The resulting 3D models were analyzed using CT software (Zeiss, Jena, Germany), which allowed for estimation of the total porosity due to the usage of binarization threshold and defect removal functions. The imaging resolution was around 20 µm.

### 2.8. Differential Scanning Calorimetry (DSC)

DSC was performed to determine the temperatures and thermal effects of phase changes using a DSC Q1000 device (TA Instruments, New Castle, DE, USA). Foam samples were placed in hermetic aluminum pans, which were initially cooled down to −90 °C, heated to 250 °C at a rate of 10 °C/min (first heating cycle), cooled down again to −90 °C at a rate of 5 °C/min, and reheated back to 250 °C at a rate of 10 °C/min (second heating cycle). The results were analyzed with Universal Analysis 2000 ver.4.5A software (TA Instruments).

### 2.9. Thermogravimetric Analysis (TGA)

Thermogravimetric analysis was done using a TGA Q500 device (TA Instruments). Samples were tested under an air atmosphere upon heating from ambient temperature to 700 °C at a heating rate of 10 °C/min. The obtained data were analyzed with Universal Analysis 2000 ver.4.5A software (TA Instruments).

### 2.10. Elastic Recovery Time and Rebound Resilience

Elastic recovery time was measured upon the release of a 100 mm × 100 mm × 100 mm sample compressed by 90% for 1 min at ambient temperature; the time was taken with an accuracy of 1 s using an electronic stopwatch. Resilience was determined according to the standard EN ISO 8307 [14]. A steel ball with a diameter of 16 mm was dropped from a height of 50 cm onto a 100 × 100 × 100 mm sample cut from the inner part of a foam element. The height of the rebound was measured using frame-by-frame video analysis.

### 2.11. Compression Set, Compression Stress Value

The compression set was determined according to the standard EN ISO 1856 [15]. Compression of 50% and 90% was applied for 22 h at 70 °C to 50 mm × 50 mm × 25 mm samples in a direction parallel to foam growth. The foam compression process on the testing machine was assessed. The samples were compressed by 60% of their height. The value of compressive stresses during loading and unloading was obtained, thus obtaining a hysteresis loop. Each sample was squeezed 4 times. The hardness of the foam (stress at 40% height of the sample) was determined based on the compression test.

### 2.12. Sweat Uptake

The uptake of artificial sweat with acidic and alkaline pH was measured for each foam variant. Samples weighing 1 g were first dried under vacuum for 24 h at 70 °C and weighed with an accuracy of 0.1 mg to assess dry foam weight (W_d_) using a WPA 180/C/1 analytical balance (Radwag, Poland). Each sample was then soaked in sweat solution for 8 h. Excess fluid was removed from the sample exterior by placing it on fresh filter paper (Thermo Fisher Scientific) for 1 min before weighing to determine the equilibrium swelling weight (W_s_). The equilibrium weight swelling ratio (Q) was calculated as the equilibrium swelling weight divided by the dry foam weight. The sweat solutions were prepared according to ISO 105-E04:2013 [16].

## 3. Results and Discussion

The performance features of foams are dependent on the foam material’s chemical structure and its pore structure. Therefore, Section 3.1, Section 3.2, Section 3.3, Section 3.4, Section 3.5, Section 3.6, Section 3.7 and Section 3.8 describe the material and pore structure characteristics of the foams. The following sections (Section 3.9, Section 3.10 and Section 3.11) describe the performance characteristics of these foams that will affect the comfort of prostheses with liners made from these foams.

### 3.1. Synthesis Parameters

The cream time is defined as the time that the mixture needs after mixing polyol with isocyanate until the reaction starts. All samples appeared as creamy mixtures short after spilling the mixture into an open mold. Rise time is the time at which the foam stops expanding. Gel time is the time at which long-chain polymer has formed and is noticeable as a tacking characteristic of the mixture. The measurements are put below in Table 3.

It can be observed that the rising and gel time of the foams increases with the INCO index. The addition of water also extends rising and gel time. Introduction of hydroxyl groups (–OH) from water prioritizes reaction of isocyanate group (–NCO) with H_2_O faster than –OH from the polyol due to –NCO mobility and accessibility of –NCO to H_2_O.

Based on the analysis of the determined parameters of the synthesis process, it is possible to design the course of the synthesis of foams in closed molds. The molding in closed molds requires longer rising times to be able to fill the mold. A desirable feature is the shortest possible gelation time to minimize the time required to de-mold the product. Considering the manufacturing process of insoles and liners, as well as other materials for orthoses, the most suitable parametric were obtained for the foams: VLux_2_0.7, VLux_2_0.8, VLux_2_0.9 and VLux_3_0.7, VLux_3_0.8, VLux_3_0.9

### 3.2. Apparent Density

**Figure 2:**
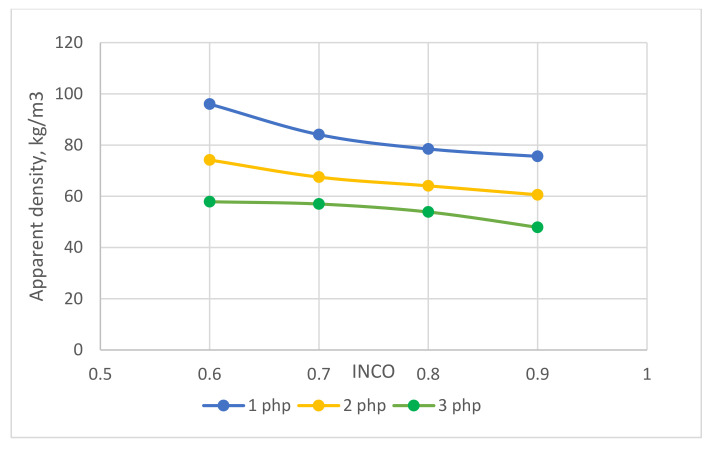
The apparent density of manufactured foams depending on the isocyanate index and water content.

The apparent density of the foams decreased with increasing INCO for the VLux_1 series of foams in the range from 96 to 76 kg/m^3^, for the VLux_2 series of foams from 74 to 60 kg/m^3^, and the VLux_3 series of foams from 58 to 48 kg/m^3^ (Table 3). Based on the given data in Figure 2, it can be combined that the genesis of apparent changes is similar.

**Figure 3:**
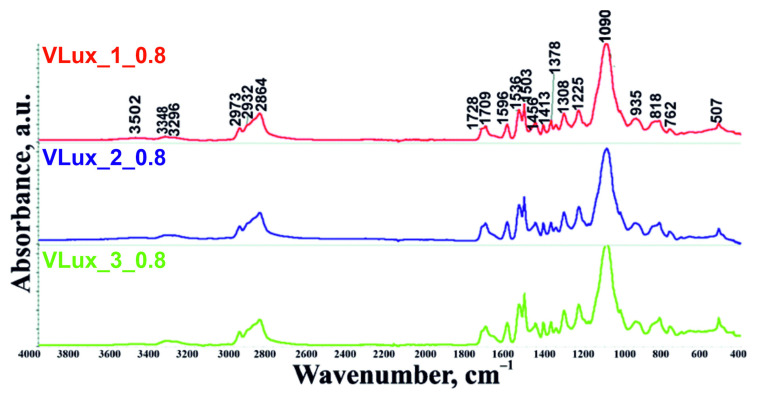
Comparison of ATR-FTIR spectra for foams with different water content at INCO = 0.8.

The decrease in apparent density with the increase in INCO is probably the result of an increase in their porosity. Such a trend of changes in porosity with an increase in INCO was presented in Krebs and Hubel [16]. Based on the data presented in Figure 3, it can also be concluded that with the same INCO, the density of the foams decreases with the increase of the water content in the foams. In this case, a decrease in apparent density is also associated with an increase in the porosity of the foams. Water is a chemical blowing agent; the more it is, the greater the porosity of the foams [17]. According to Szczepkowski et al. [18], viscoelastic foams with INCO ranging from 0.8 to 1.0 have an apparent density in the range of 43–65 kg/m^3^, while the foams described by Rajan et al. [19], which were synthesized from methylene diphenyl diisocyanate (MDI) at INCO in the range of 0.5–1.0. have a density of approx. 65–70 kg/m^3^. An apparent density of 64.7 kg/m^3^ characterized the viscoelastic foam analyzed by Kumar B. et al. [20].

During the use of orthopaedic aids, it is beneficial for their users to keep their weight as low as possible, which is achieved in materials with a lower apparent density. In the case of the tested Viscoelastic Polyurethane (VEPUR), this is a series containing 3 php of water.

### 3.3. Fourier Transform Infrared Spectroscopy (FTIR)

Tested foams were synthesized using a chemical blowing agent (i.e., water) that reacts with isocyanate monomers to produce carbon dioxide [17]. Figure 3 shows exemplary Attenuated Total Reflectance (ATR)-FTIR spectra of the tested foams, in Table S1 (Supplementary Data) summarizes bonds identified in examined foams.

FT-IR analysis allowed detecting and identifying bonds and functional groups present in the studied samples of open-cell viscoelastic polyurethane foams.

The foams were made at different isocyanate indexes (0.6–0.9); no peak was observed in the spectra in the 2270–2240 cm^−1^ band from the NCO group [21,22], indicating that all NCO groups have reacted. In the wavenumber range 3500–3000 cm^−1^, there are two bands associated with the reaction substrates. The band in the region 3345–3306 cm^−1^ originates from symmetric and asymmetric stretching vibrations attributed to the N–H bond [21,23]. Another band near wave number 3502 cm^−1^ originates from the –OH group from water and unreacted polyols. The band at wavenumber 3120 cm^−1^ corresponds to the stretching of CH bonds from aromatic groups. The spectra at wave number 2970 cm^−1^ and 2867 cm^−1^ originate from symmetric and asymmetric stretching vibrations within –CH_2_ groups in soft segments formed from polyols, respectively [21,22]. Bands originating from C=O stretching vibrations in urethane groups were observed in all analyzed samples [24]. The lower frequency band is the bound groups (1708 cm^−1^) [25], while the higher frequency band is the unbound urethane groups (1724 cm^−1^) [25]. The waveband spectrum at 1640–1690 cm^−1^ is associated with the stretching vibrations of the C=O bonds of the urea groups. The spectrum band at 1537–1506 cm^−1^ originates from bending vibrations of the NH bonds and stretching vibrations of the CN bonds in the urethane groups. Vibrations of the same bonds appear at 1460–1450 cm^−1^, but they originate from urea groups. The peaks appearing at 1373 cm^−1^ and 1412 cm^−1^ are related to bending and stretching vibrations of CH_2_ groups. The peaks 1306–1304 cm^−1^ are related to the stretching vibrations of the CN bonds. Bending vibrations of δ bonds occurring in the C–O group are detected on the highest peak of the whole spectrum, at 1090–1086 cm^−1^ [23]; this is the band occurring in polyols that form the soft phase of foams. On the spectra, bands at 765 cm^−1^ and 818 cm^−1^ related to CH bond vibrations in aromatic rings were also found [23]. Vibrations originating from aromatic rings in isocyanates occur at 1596 cm^−1^, allowing the comparison of samples’ spectra with the same isocyanate index and their quantitative analysis [21].

**Figure 4:**
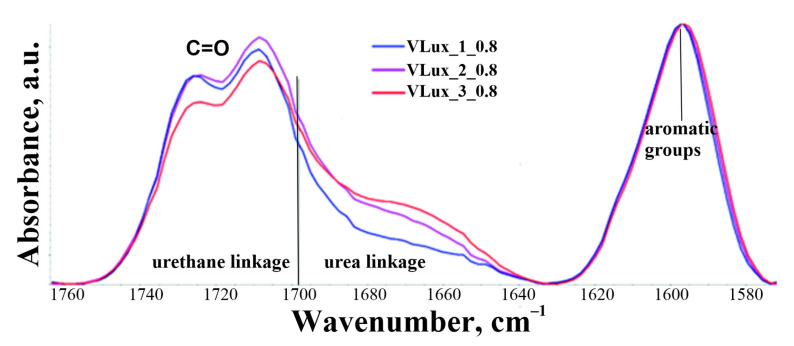
Comparison of ATR-FTIR spectra in the range 1760–1580 cm^−1^ for foams with different water content at INCO = 0.8.

Figure 4 shows the spectrum in the 1760–1580 cm^−1^ range of foams made at INCO = 0.8 containing various amounts of H_2_O, calibrated according to the 1596 cm^−1^ band.

In the range of 1698–1630 cm^−1^ there are bands from vibrations of carbonyl groups in urea groups and the range of 1750–1700 cm^−1^ bands from vibrations of carbonyl groups in urethane groups. From the graph in Figure 4, it can be concluded that the area under the curve from the vibration of the groups in the urea groupings increases with increasing water content in the blends made at the same isocyanate index. The area under the curve coming from groups in urethane groupings also changes.

**Figure 5:**
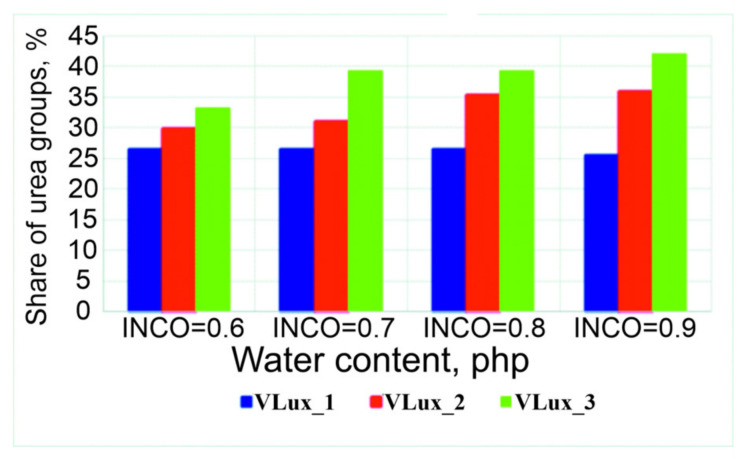
Change in the share of urea groups in the tested foams.

**Figure 6:**
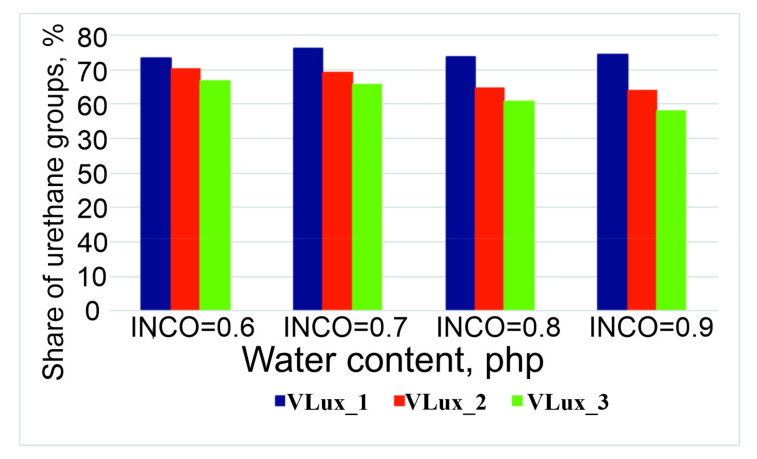
Change in the share of urethane groups in the tested foams.

Therefore, a quantitative analysis of the spectra was performed, and the results of this analysis are summarized in Figure 5 and Figure 6. The error in the analysis of the participation of individual groups was 2%. Increasing the water content in foams with the same INCO causes an increase in the share of urea bonds in the foams and a decrease in the share of urethane bonds. With a higher INCO, a higher proportion of urea groups in the resulting foams is observed.

### 3.4. Scanning Electron Microscopy (SEM)

**Figure 7:**
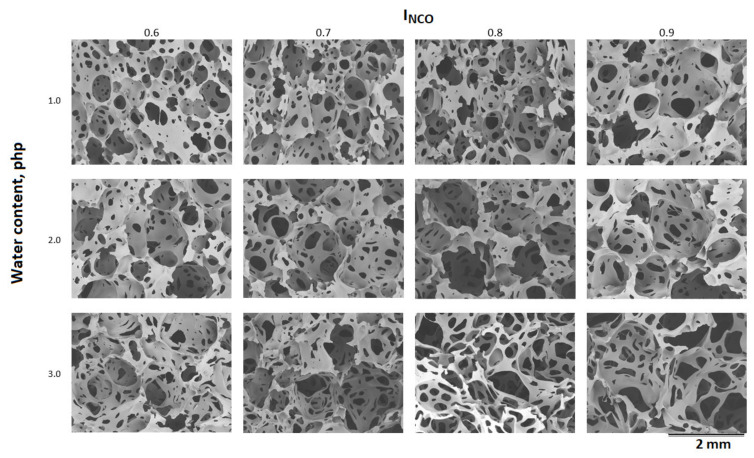
SEM images of manufactured foams with their water content and INCO index.

To evaluate differences in porosity, image analysis of the foams was performed using SEM. Figure 7 presents the microstructure of manufactured foams, with indications of INCO index and blowing agent content in the formula. The results of the quantitative analysis of SEM images are summarized in Table 4.

Based on the SEM images, it can be concluded that the more water in the foams formula, the larger the pore size in foams made with the same isocyanate index. The pore size also increases with increasing isocyanate index in foams made with the same amount of water. Water is a chemical blowing agent in the produced foams. As more water is added to the foams, the amount of CO_2_ formed due to the reaction of water with isocyanate increases. Based on the SEM images, it can be seen that in foams made with INCO = 0.9, the pore wall thickness increases compared to foams made with INCO = 0.8. Their quantitative analysis was performed to confirm the qualitative analysis based on SEM images. Its results are summarized in Table 4.

For the VLux_1 series foams, the mean pore diameter is 480–590 µm, and its spread in the range of 260–360 µm, the pore anisotropy index is about 1.43. In the VLux_2 series foams, the diameter d2 is in the range 540–610 µm, and its spread in the range of 350–490 µm), the AI pore varies in the range 1.43–1.52 µm. VLux_3 series foams had the diameter d2 in the range of 540–700 µm, and it is dispersed in the range of 420–520 µm, the AI time varies in the range of 1.48–1.58 µm. The more water the foams contain, the larger the pore size and size distribution. There is a tendency to increase the size of the pores and their size dispersion with increasing INCO. Only the VLux_1 and VLux_2 foams deviate from this trend. An increase in water content and an increase in INCO causes an increase in the foam pore anisotropy index. It can be concluded that the smaller the INCO, the greater the uniformity of the pore size.

### 3.5. Microtomography (µCT)

**Figure 8:**
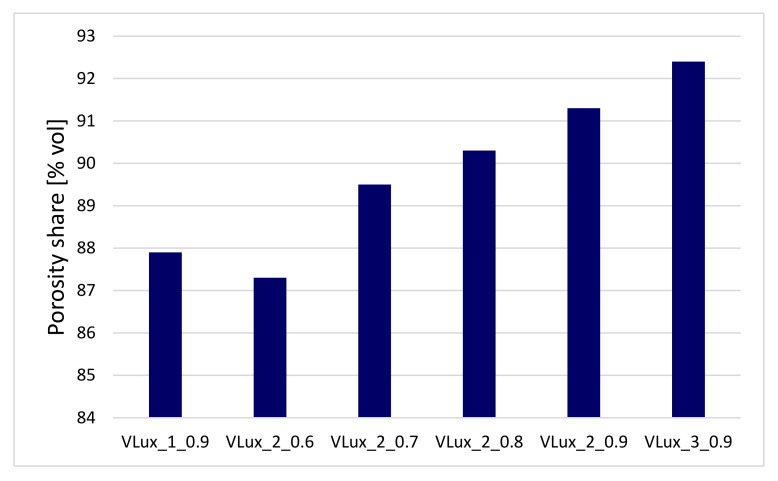
The results of the total porosity analysis of foams with different water content performed at INCO = 0.9 and of foams with different INCO containing 2 php of water were obtained during the analysis with the use of µCT.

Tests with the use of microtomography were performed only for selected foams. Foams were selected to determine the effect of the amount of water at the same INCO and the effect of INCO at the same water content (Figure 8).

The porosity of the tested materials was in the range of 87–92% vol. The foam described by Kumar et al. [20] had a slightly higher porosity of 94% vol., when the water content of the foams with the same isocyanate index increased in the range 88–92% vol.

It was observed that the porosity increased with the isocyanate index for foams made with 2 php of water. Tested foams, which is the result of an increase in the proportion of water reacting with the isocyanate, which confirms the increase in the proportion of urea bonds in polyurethane foams (Figure 5).

**Figure 9:**
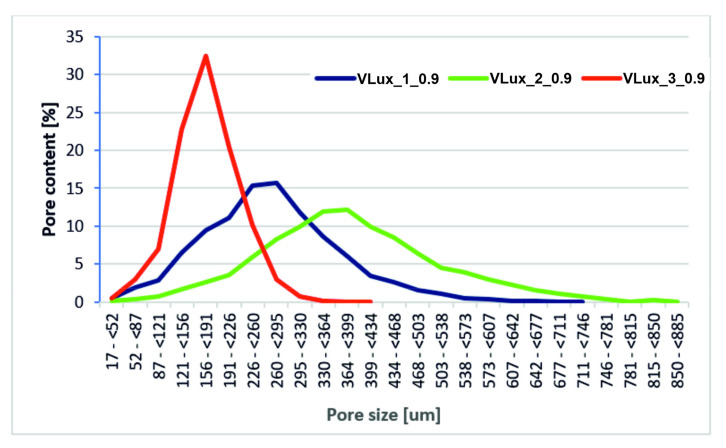
The results of the pore size analysis of foams with different water content performed at INCO = 0.9 were obtained during the analysis with the use of µCT.

**Figure 10:**
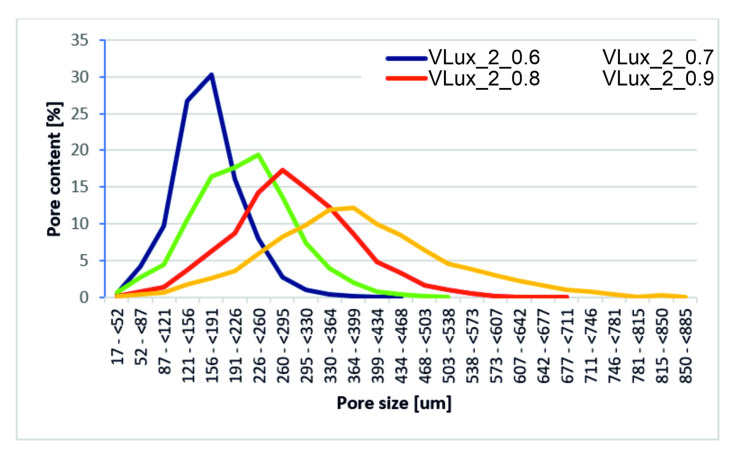
The results of the pore size analysis of foams with different INCO containing 2 php of water were obtained during the analysis using µCT.

In the tested foams, H_2_O is used as a chemical blowing agent. The pore size rises along with increasing its amount. In addition to porosity, the pore structure of these foams also changes. The pores of the VLux_1_0.9 foam are up to approx. 750 µm, and the maximum pore diameter is approx. 300 µm, the pores of the VLux_2_0.9 foam vary in a wider range and are up to 890 µm with a maximum pore diameter of approx. 400 µm (Figure 9). However, in the VLux_3_0.9 foam, the pores have a different pore size distribution than the VLux_1_0.9 and VLux_2_0.9 foams, the pores have diameters of up to approx. 430 µm with a maximum pore diameter of approx. 190 µm. In PUR foams, the pore structure also changes with the change of INCO [16], as is the case with the analyzed foams (Figure 10). In the case of VLux_2 series foams, with the change of INCO, the maximum pore diameter increases from approx. 190 µm for foam with INCO = 0.6 to approx. 320 µm for foam with INCO. Increasing INCO is also associated with increasing the width of the pore size distribution. The analysis results indicate that the degree of porosity increases with increasing cell size for foams produced using an increasing proportion of isocyanate. The increase in foam porosity with increasing cell size was also observed for foams produced using water in the proportion of 1 php and 2 php. The application of water in the proportion of 3 php caused a decrease in the cell size, but the porosity increased, indicating that the pores and ribs walls were very thin.

### 3.6. Differential Scanning Calorimetry (DSC)

From the DSC thermograms of the investigated samples, two glass transition temperatures T_g1_ and T_g2_ of the soft phase, two temperatures of the transition related to the ordered hard phase (T_t1_ and T_t2_), and the enthalpy of these transitions (ΔH) were determined. The heat capacity at the glass transition (C_P1_, C_P2_) was also determined.

**Figure 11:**
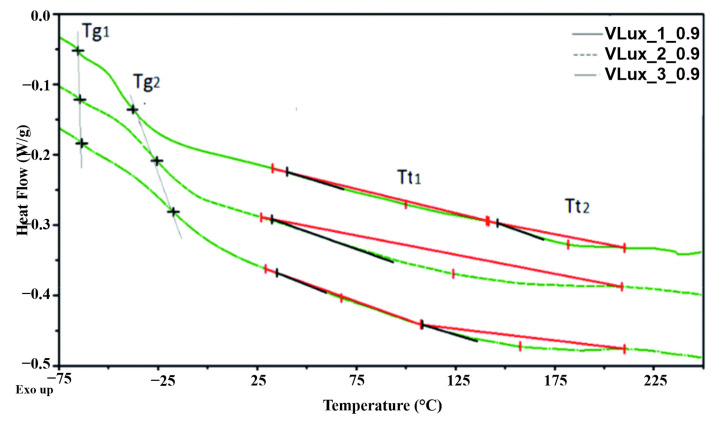
DSC thermograms were obtained in the first heating cycle for foams with different water content and INCO = 0.9.

The pictures (Figure 11 and Figure S1 (Supplementary Data)) show exemplary DSC thermograms of the tested materials and determinations of parameters determined based on these curves.

The parameters determined based on the curves obtained during DSC measurements are summarized in Table 5 and Table S3 (Supplementary Data).

Viscoelastic polyurethane (VEPUR) is made of flexible segments with different chemical structures. Therefore, there are two glass transition temperatures (T_g1_, T_g2_); the first is associated with the soft phase formed by segments of greater flexibility, and the second one with less flexibility. Increasing the water content in a formulation of foams made with the same INCO leads to more rigid segments containing urea moiety. Figure 11 and Table 5 show that both T_g1_ and T_g2_ increase with increasing water in the foam recipe. Much more significant changes in T_g2_ were observed compared to changes in T_g1_. This indicates that considerably more urea-type stiffening rigid segments are formed in the soft phase formed by flexible segments of the second type. Both T_g1_ and T_g2_ increase with increasing INCO, which is a result of limiting the mobility of flexible segments caused by an increase in the number of rigid segments in foam macromolecules [26]. In the DSC curves of all foams, the thermal effects associated with the glass transition in the soft phase described by T_g1_ and T_g2_ are visible. The lower heat effect (ΔC_p_) associated with the glass transition in the soft phase indicates less mobility of the segments making up this phase [27].

Based on the research results presented in [28], classic flexible foams made of TDI isocyanate are characterized by a T_g_ of approx. −60 °C ÷ −40 °C, and viscoelastic foams of approx. −10 °C ÷ 20 °C. In the case of viscoelastic foams made of MDI isocyanate, in the previously presented studies, it was T_g_ = −53 °C ÷ −16 °C [29] and T_g_ = −64 °C [30]. There are two glass transition T_g_ in the tested foams: T_g1_ = −65 °C ÷ −62 °C and T_g2_ = −48 °C ÷ −6 °C, so they are foams with characteristics intermediate between classic flexible foams and viscoelastic foams.

In the DSC thermograms of the foams, two changes related to the order change in the hard phase of the foams were observed. These are endothermic changes described by the temperature of these transformations T_t1_ and T_t2_ and, respectively, the enthalpy of these changes (ΔH_m1_ and ΔH_m2_). Based on model studies of thermoplastic elastomers [31], it was found that these transformations are the result of the disintegration of ordered structures composed of a population of rigid segments of similar length. For the tested model, polyurethane elastomers made of MDI and 1,4-butanediol, five endotherms were identified: 100–180 °C, 190–210 °C, 211–217 °C, 222–230 °C.

Two endothermic changes occur only in some of the foams. In foams with VLux_1_0.8 and VLux_1_0.9 and VLux_2_0.6, VLux_2_0.7 and VLux_1_0.8, there is only one hard phase transformation. It is a transformation with a much greater enthalpy than other foams, indicating that more rigid segments were organized in their hard phase. Probably the occurrence of one transformation means that for the VLux_2 series, no increase in the stiffness of the flexible segments constituting the soft phase of the foams from this series was observed (which was observed by analyzing the change in ΔC_p_).

The differences in the temperature T_t1_ and T_t2_ indicate that the foams form rigid segments with a different chemical structure and/or the length of these segments. The foams create segments with a transformation temperature T_t1_ of approx. 66 ± 4 °C, 100 °C and 122 ± 4 °C and T_t2_ of approx. 133 ± 4 °C, 154 ± 4 °C and approx. 183 °C. Based on the results presented based on model tests of thermoplastic elastomers, it can be concluded that rigid segments in foams are made of one or two isocyanate molecules (in elastomers T_t_ for these transformations is within the range of 50–70 °C and 100–180 °C) [31]. In polyurethane foams, these segments also differ in the type of bonds present in the rigid segments. Rigid segments are created in foams containing both urethane and urea bonds.

The formation of two types of rigid segments was observed in the VLux_3 series. The first one for the whole series has similar characteristics. Their T_t1_ is in the range of 63–67 °C and the enthalpy of conversion ∆H_t1_ varies in the range of 0.5–1.2 J/g. It is observed that T_t1_ in this series of foams decreases and ∆H_t1_ increases with increasing the proportion of urea groups in their rigid segments. For three of the VLux_2 series foams, rigid segments with T_t1_ around 120 °C were observed, whose ∆H_t1_ decreases with increasing the proportion of urea groups.

Such changes in the characteristics of rigid segments with different chemical structure and/or length indicate that the proportion of urea groups influences the content of hydrogen bonds connecting rigid segments, which can be bonded with ΔH_t_. In the case of shorter segments with T_t1_ about 65 °C the formation of hydrogen bonds is easier and therefore, their ΔH increases with an increasing proportion of urea groups. For longer rigid segments, their ordering is more difficult; therefore, with increasing the proportion of urea groups ΔH_t_, the amount of hydrogen bonds formed of this transformation decreases.

### 3.7. Thermogravimetric Analysis (TGA)

**Figure 12:**
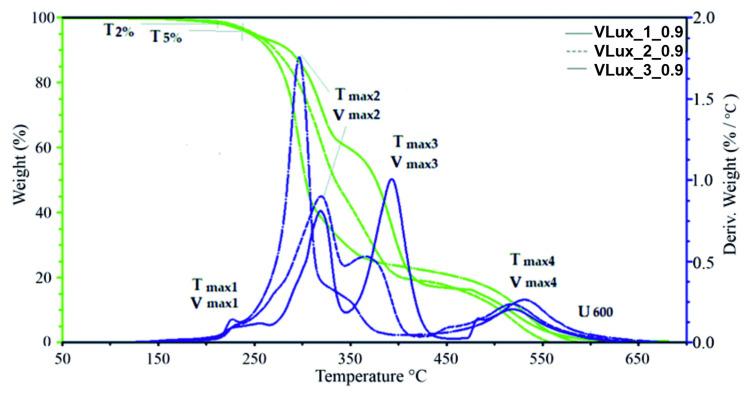
Results of TGA analysis and parameters description for foams with INCO = 0.9 and different water content.

The analysis of the obtained mass change curves (TG) carried out in the Universal Analysis V4.5A software made it possible to determine the temperature at which there was a mass loss of the sample amounting to 2% and 5% (T_2%_, T_5%_), as well as the residual mass at 600 °C (U_600_). On the other hand, from the mass change derivative curves (DTG), the temperature and the maximum velocity in the subsequent stages of degradation (T_max1_, V_max1_, T_max2_, V_max2_, T_max3_, V_max3_, T_max4_ and V_max4_, as well as weight loss in these stages (Δm_1_, Δm_2_, Δm_3_, Δm_4_) were determined. Figure 12 shows exemplary results obtained during the TGA analysis of foams made at INCO = 0.9 with different water content in the recipe, with marked parameters determined on their basis.

**Figure 13:**
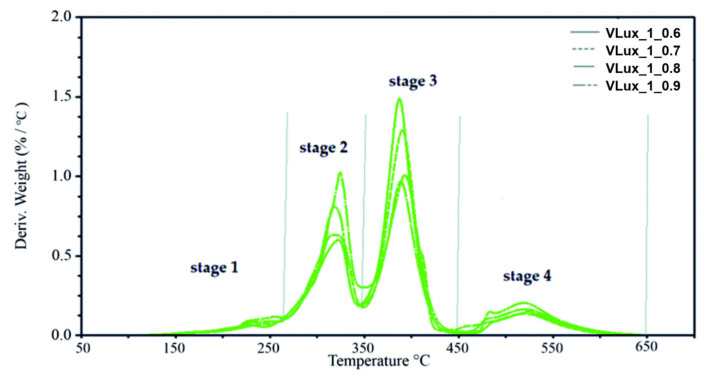
Results of TGA analysis for foams with 1 php water and different INCO.

The course of TG and DTG curves for foams with different water content in the recipe differs significantly in the range of 200–550 °C (Figure 12). This is the range in which the hard and soft phases of the foams decompose; similarly, when we analyze the influence of INCO on the thermal stability of foams (Figure 12 and Figure S2 (Supplementary Data)) at 350–450 °C, the flexible segments forming the soft phase are unfolded, while at higher temperatures, the ring structures found in the soft and hard phases of the foams are decomposed. The course of DTG curves obtained for a series of foams made with different INCO and a different amount of water is summarized in Figure 13 and Figure S2 (Supplementary Data).

In the case of foams containing 1 php of water, the DTG curves have a similar course; they differ significantly in the speed of subsequent stages of degradation (Figure 13). In the case of the VLux_2 series, the DTG curves differ not only in the degradation rate in the subsequent decomposition stages but also in the temperature at which this rate is achieved. In the VLux_3 series, foams also differ in the number of successive stages of degradation (Figure S2, Supplementary Data).

The parameters determined based on the results of the TGA analysis are presented in Table S2 (Supplementary Data).

In most of the analyzed foams, the occurrence of four stages of degradation was observed. However, for the VLux_3_0.7, VLux_3_0.8 and VLux_3_0.9 foams, there are only three degradation stages, stages 2 and 3 are not separated, which indicates that there was no separation of the hard and soft phases of these foams.

The first stage of degradation takes place with the maximum degradation rate in the temperature range of 225 ± 4 °C and 263 ± 4 °C and the maximum degradation rate in the range of 0.07 ÷ 0.22%/°C. In most foams, the rate of degradation increases with increasing INCO. During this stage, 3–10% of the mass is lost. It can be assumed that in step 1, short segments of the rigid foams decompose. In the range of 260–350 °C, the segments of rigid foams of a different structure than in stage 1 are degraded.

At this stage, there is a tendency to increase the degradation rate with increasing INCO and higher water content in the foam recipe. At this stage, VLux_1 series foams lose 30–37% of the mass, and in the VLux_2 series foams, 30–49% of the mass is lost. In stage 3, in the temperature range of 350–450 °C, the segments that make up the soft phase of the foams are degraded. The VLux_1 series loses 52–40% of the weight, the VLux_2 series loses 43–27% of the weight, and the VLux_3 series loses 73–69% of the weight. The amount of weight loss in this step and the maximum rate of degradation decreases with increasing INCO of the foams. The fourth stage of decomposition is related to the degradation of the degradation residues in the previous stages containing a significant amount of aromatic compounds. It takes place in the temperature range of approx. 420–650 °C. At this stage, approx. 10–19% of the mass is lost with increasing INCO; the mass decreasing in this stage increases. At this stage, the maximum degradation rate ranges from 0.14–0.27%/°C and increases with increasing INCO in each series of foams. At this stage, the maximum degradation rate is reached in the temperature range of 517–531 °C.

Changes in the maximum degradation rate in the first stage of decomposition (V_max1_) were analyzed, depending on the urea groups’ share changes. It was found that with increasing the share of urea bonds, there is a tendency to increase V_max1_, i.e., decrease in thermal resistance of these materials. This characteristic of foams may be necessary when multiple sterilizations are expected in some of their applications. The reduction of thermal resistance in the first stage of degradation may impact the reduction of the number of sterilization processes that can be carried out for foams with higher V_max1_.

Murray et al. [32] studies have shown that commercial polyurethane (Pellethane 2363 90A) chemical structure was changing after sterilization via electron beam (e-beam), but Briggs et al. [33] prove with their own foam formula that e-beam sterilization dosages between 21 and 30 kGy do not influence their investigated samples degradation profile of these polyurethane foams.

### 3.8. Gel Fraction

**Figure 14:**
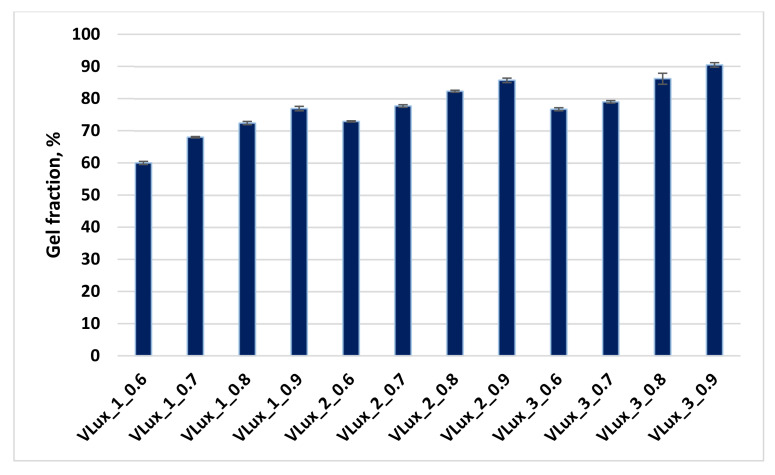
Gel fraction analysis of analyzed foams.

The analyzed foams contained 60–90 wt.% of the gel fraction. In each series of foams with different water content, the gel fraction content increased with increasing INCO. Increasing the proportion of the gel fraction may result from increasing the number of urethane groups formed in the macromolecules of the foams and increasing their INCO. On the other hand, in the case of foams made with the same INCO and different in the content of H_2_O, the increase in the gel fraction content results from the increase in the urea content. The results are summarized in Figure 14.

After the gel fraction analysis, the foams were subjected to thermal analysis. Sample results of TGA analysis of foams before and after exposure to dimethylformamide (DMF) (Figure S4 Supplementary Data).

The DTG curves observed three distinct degradation stages, and stage 4 is only marked in the VLux_3 series (Figure S4 Supplementary Data). The first stage is in the range of 150–260 °C, the second stage is completed at about 340 °C and the third stage is about 450 °C. In materials after the analysis in DMF, the subsequent stages of thermal degradation take place in a similar temperature range in which the process of thermal degradation of the foams is analyzed after the synthesis process takes place. In each series of foams, the successive stages of degradation differ in the maximum rate and temperature of degradation. For each of the tested materials, during the exposure to DMF, there is a loss of macromolecular fragments containing rigid segments formed from MDI, i.e., urethane and urea groups. In the course of thermal degradation, these fragments decompose in the first and second stages of decomposition. This assumption is confirmed by the lack or weakly marked fourth degradation stage, which decomposes ring compounds.

After exposure to DMF, DSC thermograms clearly show the glass transition temperature of the soft phase and the endothermic peak associated with the change of order in the hard phase (Figure S3 Supplementary Data). This results from a reduction in the share of rigid segments limiting the rotation of flexible segments [34]. The T_g_ and endothermic peak analysis results after exposure to DMF are presented in Table S3 (Supplementary Data).

### 3.9. Elastic Recovery Time and Rebound Resilience

**Figure 15:**
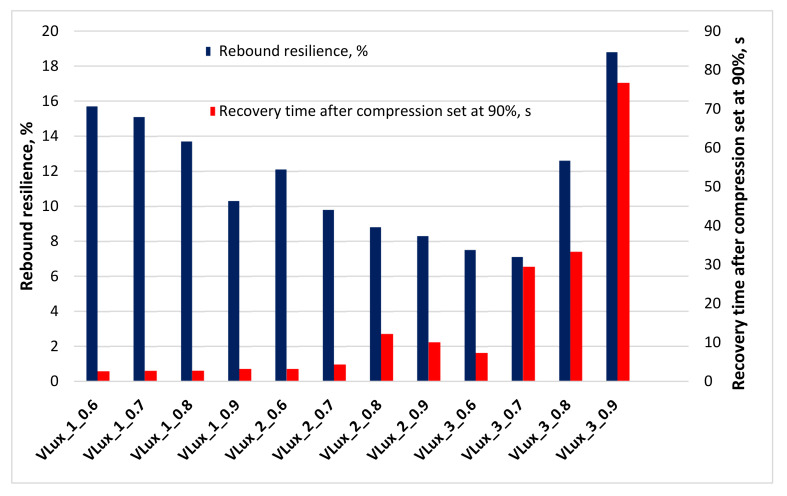
Recovery time and rebound resilience of analyzed foams.

The suitability of viscoelastic foams for use as auxiliary elements in orthopedics will be determined by their slow recovery after deformation and low resilience, which distinguish these foams from the group of flexible foams. The change in VEPUR deformation after the load is removed results in resilient force. Additionally, the typical VEPUR slow recovery after deformation foams is achieved by balancing this force by reversed effects. This effect results from three phenomena: the pneumatic effect, the adhesion effect and the relaxation effect. The level of resilience force, otherwise known as the network resilience effect in foams, depends on the structure of their macromolecules and their arrangement and slightly depends on the temperature and cellular structure of the foams. The relaxation effect also depends on the structure of macromolecules and their order, but the temperature of use significantly influences its level. The adhesion effect depends on the structure at the molecular and macroscopic levels and temperature. The cellular structure of the foams significantly influences this effect. On the other hand, the pneumatic effect is mainly influenced by the macroscopic structure of foams [17]. The description of the recovery time analysis results is presented in Figure 15.

The recovery time of the foams after 90% deformation increased with increasing water content in the formulation and with increasing INCO (Figure 15). Increasing the INCO and the amount of water in the foams leads to an increase in the number of rigid segments, which results in reduced mobility of the flexible segments. The reduced mobility of soft segments directly affects the polyurethane foam recovery time [35].

In materials for orthopedic components, the recovery time must not be too short or too long to perform their function well. From the point of view of these applications, the materials studied are interesting: VLux_2_0.8, VLux_2_0.9 and VLux_3_0.6 foams.

The work of Okrasa et al. [29] also presents formulations of foams with similar elastic recovery times. For materials usually used to manufacture liners, i.e., silicone, polyurethane [36], recovery time after compression is not determined.

All analyzed foams have a rebound lower than 20%, typical for VEPUR foams.

The low elasticity of VEPUR foams and their ability to conform to element shape and release increased pressure between the skin and bony elements means that viscoelastic foams reduce skin damage [34].

The low elastic deformation of the foams will favor the damping of vibrations transmitted from the prosthesis to the limb. It was observed that with the increase in INCO, the elastic rebound decreased for the foams of the VLux_1 and VLux_2 series. The reason for reducing the elastic rebound with increasing INCO is probably the increased relaxation effect of foams resulting from changes in the structure of their macromolecules. On the other hand, for the VLux_3 series foams, the elastic rebound increases with increasing INCO. As a result of the organoleptic analysis of the foams, it was found that the adhesive effect in the VLux_3 series foams was significantly reduced. In the VLux_3 series foams, the increase in INCO causes such changes in the cell structure, which increase the reversed effects in these foams.

Low elasticity of VEPUR foams results from the summation of elasticity of segments of different lengths formed from different polyols and the level of hydrogen interactions connecting rigid segments of their macromolecules.

These features can be described by characterization with the use of DSC. The elasticity of the flexible segments is described by their C_p_ determined at T_g_ and H_t1_ and H_t2_ of the rigid segments of the order-disorder transformation. The lower the heat effect of glass transition (C_p_), the lower the mobility and, consequently, the elasticity of flexible segments [27].

Some of the foams have C_p1_ in the range of 0.05–0.06 [J/g·°C]; these foams were characterized by lower elasticity below 12%, others had higher C_p1_ 0.07 [J/g·°C] and higher elasticity >12%.

Recovery time after compression set at 90% may depend on the pore structure of foams. It was observed that the recovery time increased with increasing pore size for the VLux_1 and VLux_2 series of foams. For the VLux_1 series, the rate of this change is much lower than for the VLux_2 series. The recovery time is also affected by the anisotropy coefficient of the foam pores. For the VLux_1 series, the rate of change of recovery time with increasing anisotropy coefficient is much less than for the VLux_3 series.

Aou et al. [35] demonstrated the dependency of soft domain segmental mobility on foam recovery time. The slower was soft domain segmental mobility, the longer foam recovery time was observed. The study results also indicated that the reduction of soft segments mobility amplifies the viscous dampening of the foam.

### 3.10. Compression Set, Compression Stress Value

For the liner insert to retain its properties over extended periods of use, its low permanent deformation is essential. Compression set at 50% (22 h, 70 °C) and compression set at 90% (22 h, 70 °C) were determined for the tested foams to evaluate the permanent deformation of these materials.

The results summarized in Table 6 show that all foams after 50% deformation achieve deformation below 10%. Most foams also achieve low permanent deformation when compressed by 90%. Only the VLux_3_0.8 and VLux_3_0.9 foams exhibit large permanent deformations of more than 80%.

The hardness of the foams measured along and across the growth direction increases with increasing INCO and the increasing water content in the foams. The increase in hardness results from the increase in the number of rigid segments results from an increase in the content of rigid segments in the foams. The difference between the hardness marked in the direction of the growth of the foams and the hardness marked perpendicular to the growth direction is the result of the pore anisotropy.

Cagle et al. [36] presented studies of different groups of materials used as liners. Polyurethane liners had an average stiffness determined in compression set of 300 ± 40 kPa, silicone liners 310 ± 100 kPa and thermoplastic elastomers (TPE) liners 140 ± 30 kPa. The tested foams have significantly lower compressive strengths in the range of 0.6–1.6 kPa, so their applications as liner inserts were envisaged.

For each of the series of VLux_1 and VLux_2 foams, a tendency to increase the hardness CV40% with increasing pore size of the foams and increasing the proportion of urethane bonds in the materials studied was observed.

### 3.11. Sweat Uptake

Prosthetic insoles made of solid materials such as polyurethane, silicone or TPU are often supplemented with socks worn on the stump [37,38]. These materials cause temperatures to rise in the prosthesis socket, causing the limb stump to sweat and moisture to accumulate. Increased moisture can lead to dermatitis and infection [39].

One solution to prevent this problem is perforating materials from which liners are made [40] or SmartTemp liners [41].

Due to their porosity, the tested materials can absorb moisture and thus promote a decrease in temperature inside the prosthesis socket.

Materials presented in the study in Table 7 exhibited various sweat adsorption properties. The equilibrium swelling ratio decreased with increasing I_NCO_ and water content in the formulas. That is probably due to the more porous structure of the foams, reducing the surface area to which fluid could adhere. For most samples, swelling uptake was higher for alkaline sweat. That could be caused by the higher surface tension of alkaline sweat. The surface tension of two sweat solutions was calculated using the Pendent Drop method [42], where obtained results were 74.23 mN/m for acidic sweat and 75.03 mN/m for alkaline sweat.

The properties of the foams were analyzed. It was observed that for all series of foams, the sweat absorbency is related to their gel fraction; with increasing gel fraction of the foams, the absorbency of both types of sweat by the foams decreases.

## 4. Conclusions

There are more people after limb amputation every year, often caused by injuries and vascular diseases. Due to the ageing of the population, the number of amputations caused by vascular diseases will increase, and consequently, the need for prostheses will increase. Prostheses are to support the independence of their users, and the solutions used in their construction are to ensure the high comfort of their use. As part of the work to increase the comfort of using prostheses, viscoelastic foams as an intermediate layer between the liner and the limb was proposed.

The article presents the results of tests of twelve viscoelastic foams made with variable INCO and containing a different amount of water as a blowing agent. FTIR analysis revealed that increasing INCO and water content influences the ratio between urea and urethane groups, decreasing the share of urethane bonds.

An increase in INCO and increased water content restricts the mobility of flexible segments, which is manifested by an increase in the glass transition temperature of the soft phase. TGA analysis shows that phase structure differs in VLux_3 series foams compared to the other two series. The foams differ in their macroscopic structure, mean pore diameter, anisotropy, pore diameter distribution, and porosity, influencing their functional and mechanical properties. Increasing water content and raising the INCO index led to a higher hardness of the foams. Most foams (excluding VLux_3_0.7, VLux_3_0.8 and VLux_3_0.9 foams) have a low compression set of less than 10%.

The analysis of all the properties of the foams shows that it is preferable to prepare foams for use as auxiliary materials in orthopedics with a water content of approx. 2% and an isocyanate index of 0.8–0.9.

The concept of sweat-wicking by viscoelastic polyurethane might be an effective way to deal with an excessive sweating stump in the prosthesis, widespread in patients with elastomer liners. The fabricated material is also an innovation in preventing patients from suffering from pain caused by poor fit.

In the course of analyzes of the produced viscoelastic foams, the relationships between the chemical structure, the structure of the foams and the variable INCO of foams made with the use of MDI, as well as the variable content of water used as a chemical blowing agent in these foams, were discovered. The dependencies presented in the article will facilitate the design of new applications of viscoelastic foams.

During further work on applying the studied foams in prosthetics, tests of these materials in contact with tissues should be carried out. Future studies are going to be focused on final properties improvement. The conducted research is the first stage of work on the applications of viscoelastic foams as auxiliary materials in orthopedics.

## Figures and Tables

**Table 1:** Characteristics of polyol masterbatches.

Water Content, php	ROH (Equivalent of Hydroxyl Groups), g/mol
0.0	622.1
1.0	381.6
2.0	272.0
3.0	213.4

**Table 2:** Characteristics of manufactured polyurethane foams.

Sample Symbol	Foam System	Water Content [php]	INCO Index
RL1	VLux_1_0.6	1	0.6
RL2	VLux_1_0.7	1	0.7
RL3	VLux_1_0.8	1	0.8
RL4	VLux_1_0.9	1	0.9
RL5	VLux_2_0.6	2	0.6
RL6	VLux_2_0.7	2	0.7
RL7	VLux_2_0.8	2	0.8
RL8	VLux_2_0.9	2	0.9
RL9	VLux_3_0.6	3	0.6
RL10	VLux_3_0.7	3	0.7
RL11	VLux_3_0.8	3	0.8
RL12	VLux_3_0.9	3	0.9

**Table 3:** Characteristic time, the apparent density of manufactured foams.

Foam System	Cream Time [s]	Rising Time [s]	Gel Time [s]	Apparent Density [kg/m^3^]
VLux_1_0.6	2	110	135	96.0 ± 0.2
VLux_1_0.7	2	112	137	84.1 ± 0.1
VLux_1_0.8	2	112	140	78.5 ± 0.2
VLux_1_0.9	2	112	140	75.6 ± 0.2
VLux_2_0.6	1	90	160	74.2 ± 0.1
VLux_2_0.7	2	114	160	67.5 ± 0.2
VLux_2_0.8	3	130	225	64.1 ± 0.1
VLux_2_0.9	3	150	260	60.6 ± 0.2
VLux_3_0.6	2	90	120	57.9 ± 0.2
VLux_3_0.7	2	100	135	57.0 ± 0.3
VLux_3_0.8	2	130	200	53.9 ± 0.1
VLux_3_0.9	3	140	220	47.9 ± 0.1

**Table 4:** Results of quantitative SEM images analysis.

Foam System	Mean Pore Equivalent Diameter d_2_ [µm]	Pore Anizotropy Index AI [a.u.]
VLux_1_0.6	481 ± 366	1.41 ± 0.33
VLux_1_0.7	503 ± 253	1.42 ± 0.33
VLux_1_0.8	593 ± 321	1.44 ± 0.35
VLux_1_0.9	560 ± 322	1.44 ± 0.29
VLux_2_0.6	537 ± 338	1.45 ± 0.34
VLux_2_0.7	568 ± 352	1.40 ± 0.29
VLux_2_0.8	604 ± 486	1.44 ± 0.38
VLux_2_0.9	611 ± 432	1.52 ± 0.43
VLux_3_0.6	601 ± 510	1.48 ± 0.40
VLux_3_0.7	540 ± 424	1.52 ± 0.51
VLux_3_0.8	697 ± 504	1.55 ± 0.47
VLux_3_0.9	652 ± 515	1.58 ± 0.39

**Table 5:** Differential scanning calorimetry DSC results obtained from the first heating cycle.

Sample Symbol	T_g1_ [°C]	C_P1_ [J/g·°C]	T_g2_ [°C]	C_P2_ [J/g·°C]	T_t1_ [°C]	ΔH_1_ [J/g]	T_t2_ [°C]	ΔH_2_ [J/g]
VLux_1_0.6	−65.2	0.07	−47.6	0.44	74.4	3.7	147.8	3.8
VLux_1_0.7	−65.0	0.07	−45.8	0.43	75.0	2.1	183.0	3.9
VLux_1_0.8	−64.8	0.07	−39.9	0.47	100.2	11.1	-	-
VLux_1_0.9	−64.4	0.06	−35.7	0.46	101.4	10.5	-	-
VLux_2_0.6	−64.6	0.06	−38.4	0.45	119.4	30.0	-	-
VLux_2_0.7	−64.6	0.06	−31.1	0.48	123.7	23.9	-	-
VLux_2_0.8	−64.1	0.05	−26.1	0.50	122.2	21.5	-	-
VLux_2_0.9	−62.2	0.05	−18.9	0.56	58.7	1.1	136.8	8.4
VLux_3_0.6	−64.3	0.06	−19.8	0.44	66.2	0.5	157.9	11.0
VLux_3_0.7	−64.6	0.06	−19.3	0.48	67.0	0.6	154.5	7.4
VLux_3_0.8	−61.9	0.07	−13.7	0.59	63.7	0.9	155.8	8.1
VLux_3_0.9	−62.0	0.08	−6.3	0.69	63.3	1.2	129.3	8.3

**Table 6:** Compression set at 50% and 90% with compression stress value for different INCO and water content levels.

Sample	Compression Set at 50% (22 h, 70 °C). %	Compression Set at 90% (22 h, 70 °C). %	Hardness CV_40%_, Pa (Parallel to Foam Rise)	Hardness CV_40%_, Pa (Perpendicular to Foam Rise)
VLux_1_0.6	1 ± 2	2 ± 2	615 ± 6	541 ± 22
VLux_1_0.7	0 ± 1	1 ± 1	785 ± 27	670 ± 12
VLux_1_0.8	0 ± 0	1 ± 1	888 ± 27	806 ± 17
VLux_1_0.9	0 ± 0	1 ± 0	957 ± 18	907 ± 60
VLux_2_0.6	2 ± 4	0 ± 0	752 ± 32	712 ± 43
VLux_2_0.7	1 ± 2	1 ± 1	816 ± 48	731 ± 59
VLux_2_0.8	3 ± 2	4 ± 2	933 ± 84	863 ± 43
VLux_2_0.9	1 ± 2	1 ± 1	1208 ± 30	1133 ± 67
VLux_3_0.6	4 ± 3	0 ± 0	463 ± 37	487 ± 31
VLux_3_0.7	1 ± 1	1 ± 1	543 ± 21	517 ± 17
VLux_3_0.8	1 ± 1	84 ± 5	797 ± 20	800 ± 31
VLux_3_0.9	7 ± 1	80 ± 6	1784 ± 78	1631 ± 70

**Table 7:** Comparison of equilibrium swelling ratio for alkaline and acidic pH sweat depending on I_NCO_ and water content.

Sample	Equilibrium Swelling Ratio, g_wet_/g_dry_	
	Alkaline Sweat	Acidic Sweat
VLux_1_0.6	24.0 ± 1.7	22.9 ± 1.3
VLux_1_0.7	23.0 ± 2.4	22.4 ± 0.6
VLux_1_0.8	21.2 ± 1.6	19.8 ± 1.4
VLux_1_0.9	18.6 ± 1.9	19.5 ± 0.6
VLux_2_0.6	19.9 ± 1.5	18.4 ± 1.3
VLux_2_0.7	15.3 ± 3.9	17.3 ± 1.7
VLux_2_0.8	14.2 ± 4.0	13.9 ± 3.8
VLux_2_0.9	14.2 ± 2.6	13.7 ± 1.1
VLux_3_0.6	18.8 ± 0.7	15.6 ± 0.2
VLux_3_0.7	16.8 ± 2.6	16.0 ± 2.2
VLux_3_0.8	10.4 ± 1.3	10.0 ± 1.1
VLux_3_0.9	9.0 ± 0.8	8.6 ± 4.1

## Data Availability

Experimental methods and results are available from the authors.

## Supplementary Materials

The following supporting information can be downloaded at: https://www.mdpi.com/article/10.3390/ma15010133/s1, Figure S1: DSC derivative thermograms obtained in the first heating cycle for foams with different INCO and 1php water, Figure S2: Results of TGA analysis and parameters description for foams with different INCO: (a) series VLux_2, (b) series VLux_3, Figure S3: Thermogram DSC of polyurethane foams with INCO = 0.7 and different content of water before and after of DMF exposition, Figure S4: DTG curves of foams before and after exposure to DMF: (a) VLux_1_0.7 (b) VLux_2_0.7 and (c) VLux_3_0.7, Table S1: Summary of identified bonds present in investigated foams, Table S2: The results of TG and DTG curves of prepared foams analysis. Table S3: Differential scanning calorimetry DSC results obtained from the first heating cycle after DMF.
