# Supplementary material for: Viscoelastic Polyurethane Foams for Use as Auxiliary Materials in Orthopedics
Source: Materials (Basel). 2021 Dec 24;15(1):133. doi: 10.3390/ma15010133 (PMC8746291; doi:10.3390/ma15010133)
Supplement: Supplementary file 1 [file materials-15-00133-s001.zip › materials-1502208-supplementary.pdf]

materials

MDPI

Supplementary Materials

Viscoelastic Polyurethane Foams for Use as Auxiliary Materials in Orthopedics

Dominik Grzęda, Grzegorz Węgrzyk, Milena Leszczyńska *, Leonard Szczepkowski, Michał Gloc and Joanna Ryszkowska *

Section S1. The Synthesis Procedure.

After adding the isocyanate component to the masterbatch, the system was mixed using a high-speed stirrer (Siemens 3 MOT 1LA/080, Siemens, Munich, Germany) at 3000 rpm over 6 s. The mixture was poured into a 1500 ml open polypropylene mould. The foaming process parameters (start, rise, and gel time) were determined during the synthesis. Subsequently, the foams were cured at 70°C for 30 min and conditioned at ambient temperature for 22 h before demolding. Before testing, the samples were conditioned at ambient temperature for at least 72 h.

Section S2. The Chemical Composition of the Foams

Results of ATR-FTIR Spectra of the Tested Foams

Paragraph 3.3 shows the results of the spectroscopic analysis of the produced materials. Table S1 summarises bonds identified in examined foams.

Table S1. Summary of identified bonds present in investigated foams.

Wavenumber [cm 1]	Bond	Vibration type
3502	OH	stretching
3345–3296	NH	stretching
3120	CH (aromatic)	stretching
2970-2861	CH (methyl and methylene)	Asymmetrical and symmetrical stretching
1724–1708 (amide I band)	C=O (urethane. nonbonded and H-bonded)	stretching
1596	C=C (aromatic ring)	stretching
1537–1503 (amide II band)	C-N-H (urethane)	N–H bending + C–N stretching
1460–1450 (amide II band)	C-N-H (urea)	stretching
1412–1344	C-H (methylene)	bending and wagging
1304–1306	C-N (urethane)	stretching
1232 (amide III band)	C-O-C (ether)	asymmetrical stretching
	N-H	bending
	C-N	stretching
1178	C-H	wagging
1093–1085	C-O (urethane and ether group)	stretching
1016	C-H	symmetrical stretching
765 and 818	CH (aromatic ring)	

Section S3. The Thermal Analysis of the Foams

Thermogram DSC (Differential Scanning Calorimetry) of the Tested Foams

Section 3.6 presents the results of the DSC analysis of the produced materials, Figure S1 presents exemplary DSC thermograms of foams produced with the variable INCO (isocyanate index) in examined foams

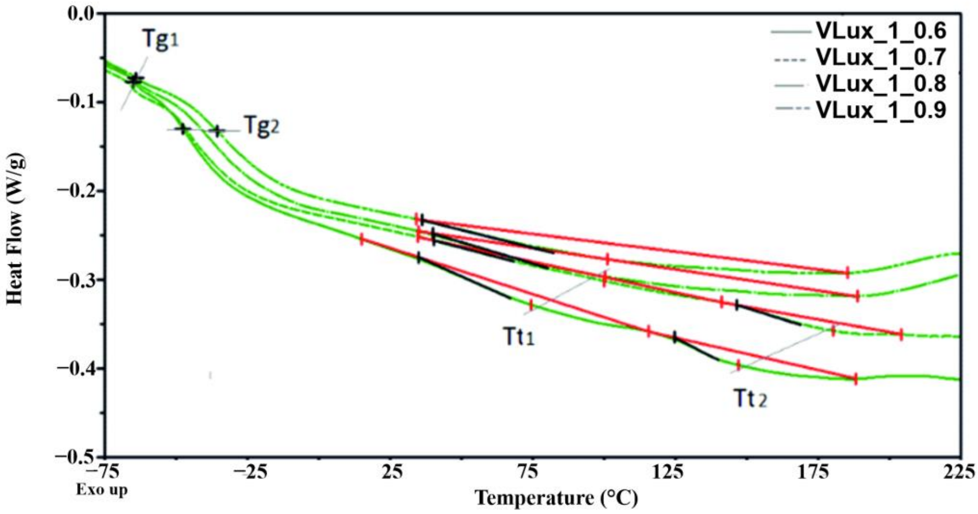

Figure S1. DSC derivative thermograms obtained in the first heating cycle for foams with different INCO and 1php water.

Section 3.7 shows the results of the TGA analysis of the produced materials produced with a variable INCO. Figure S2 presents results of TGA analysis and parameters description for foams with different INCO: (a) series VLux_2, (b) series VLux_3 and Table S2 presents the results of TG and DTG curves of prepared foams analysis.

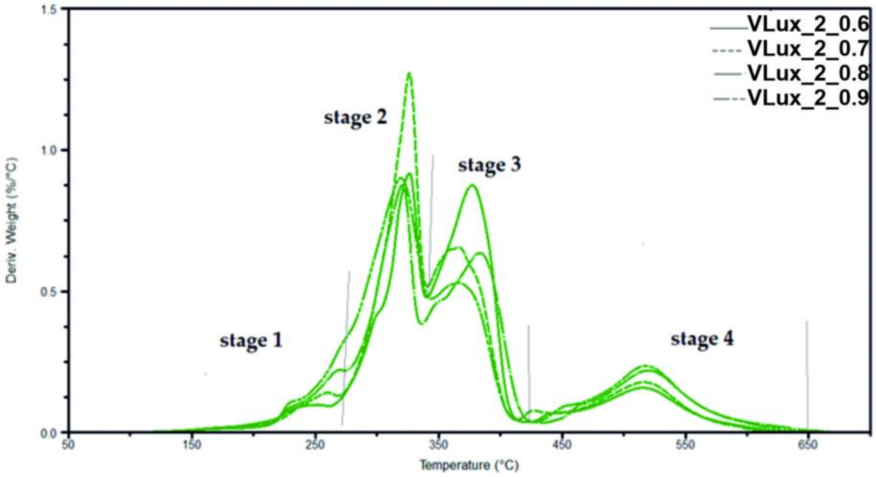

(**a**)

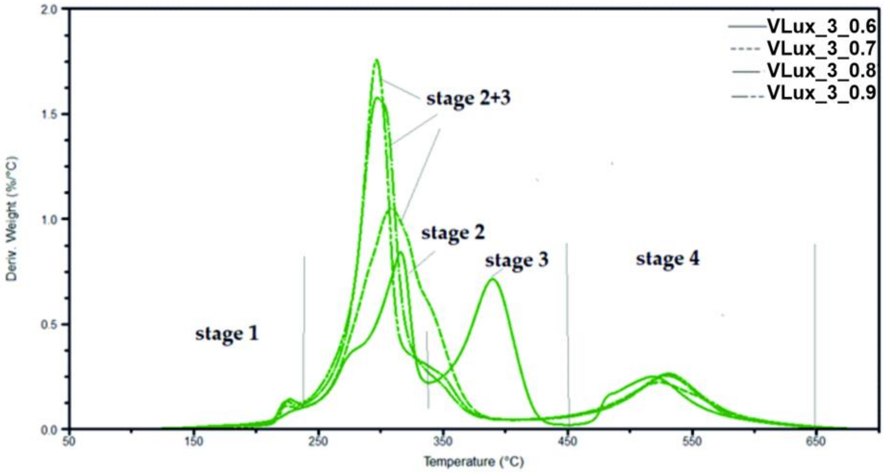

(**b**)

Figure S2. Results of TGA analysis and parameters description for foams with different INCO: (**a**) series VLux_2, (**b**) series VLux_3.

Table S2. The results of TG and DTG curves of prepared foams analysis.

**Sample Symbol**											
VLux_1				VLux_2				VLux_3			
0.6	0.7	0.8	0.9	0.6	0.7	0.8	0.9	0.6	0.7	0.8	0.9
**Results of TG and DTG Curves Analysis**											
T2% °C											
207	214	218	218	218	224	227	227	218	220	222	225
T5% °C											
257	260	255	249	254	255	253	251	249	248	246	246.
**Step 1**											
Tmax1 (the temperature. at which Vmax1 is reached), °C											
227	238	229	228	237	259	266	228	229	225	224	227
Vmax1 (maximum degradation rate of step 1), %/°C											
0.07	0.07	0.08	0.09	0.09	0.15	0.22	0.10	0.09	0.11	0.13	0.14
Δm_1, wt.%											
3.4	4.1	4.3	6.4	4.6	6.7	10.0	3.2	3.8	3.7	3.1	2.6
**Step 2**											
Tmax2 (the temperature. at which Vmax2 is reached), °C											
333	322	324	319	326	327	321	320	316	309	297	297
Vmax2 (maximum degradation rate of step 2), %/°C											
0.38	0.63	1.02	0.81	0.92	1.27	0.88	0.90	0.84	1.05	1.58	1.76
Δm_2, wt.%											
30	32	37	31	33	41	30	49	36	-	-	-
**Step 3**											
Tmax3 (the temperature. at which Vmax3 is reached), °C											
388	390	389	393	378	367	383	366	390	-	-	-
Vmax3 (maximum degradation rate of step 3), %/°C											
1.49	1.29	0.97	1.01	0.87	0.65	0.64	0.53	0.71	-	-	-
Δm_3, %											
52	48	40	43	43	33.	38	27	73	69	72	71
**Step 4**											
Tmax4 (the temperature at which Vmax4 is reached), °C											

525	521	520	520	517	517	522	517	518	525	530	531
Vmax4 (maximum degradation rate of step 4), %/°C											
0.15	0.14	0.17	0.21	0.16	0.18	0.22	0.24	0.25	0.22	0.26	0.27
Δm_4, wt.%											
10.7	11.7	13.0	14.6	14.7	17.0	18.7	18.7	17.4	18.3	17.2	17.6
U_{600}, wt.%											
1.0	1.6	1.4	0.9	1.6	0.1	0.9	0	1.1	1.6	0	0

Section 3.8 shows the results of gel fraction analysis of the produced materials with a variable INCO, Figure S3 presents results from DSC analysis before and after exposure to DMF for VLux foam sampes with INCO=0.7 and Figure S4 presents DTG curves of foams before and after exposure to DMF: (a) VLux_1_0.7, (b) VLux_2_0.7 and (c) VLux_3_0.7. Table S3 summarizes DSC results obtained from the first heating cycle after DMF.

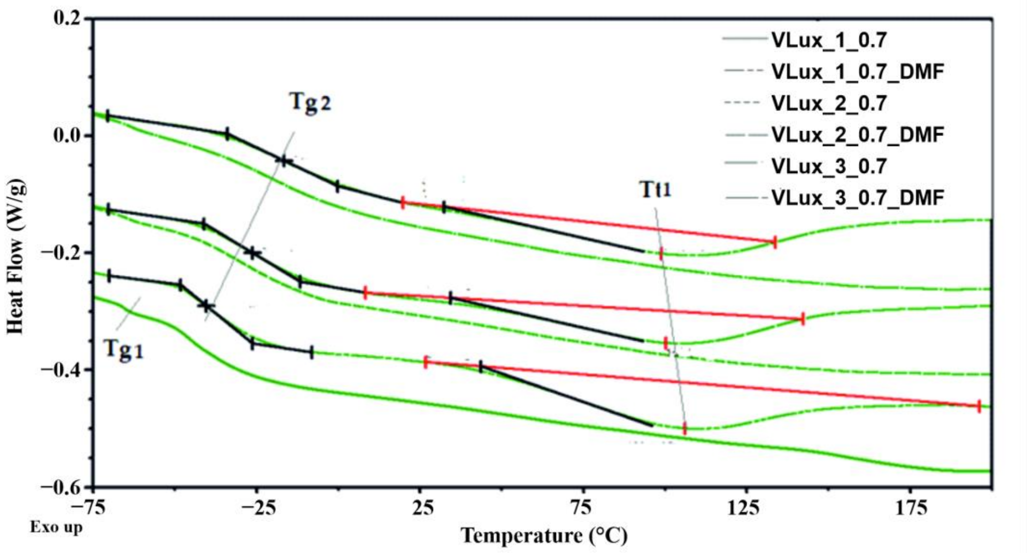

Figure S3. Thermogram DSC of polyurethane foams with INCO = 0.7 and different content of water before and after of DMF exposition.

Table S3. Differential scanning calorimetry DSC results obtained from the first heating cycle after DMF.

Sample Symbol	Tg_{2DMF} [°C]	C_{P2DMF} [J/g·°C]	T_{t2DMF} [°C]	$\Delta H_{1\Delta M\Phi}$ [J/g]
VLux_1_0.6	−45.2	0.60	100.0	38.5
VLux_1_0.7	−41.7	0.56	99.2	31.7
VLux_1_0.8	−35.9	0.58	79.5	29.6
VLux_1_0.9	−31.7	0.58	91.4	29.5
VLux_2_0.6	−35.7	0.60	85.5	25.5
VLux_2_0.7	−26.0	0.45	83.0	17.2
VLux_2_0.8	−21.1	0.43	81.9	15.5
VLux_2_0.9	−17.0	0.51	75.8	14.7
VLux_3_0.6	−20.5	0.51	89.7	6.1
VLux_3_0.7	−16.5	0.56	97.0	6.9
VLux_3_0.8	−7.2	0.52	97.2	6.6
VLux_3_0.9	1.0	0.48	65.8	4.6

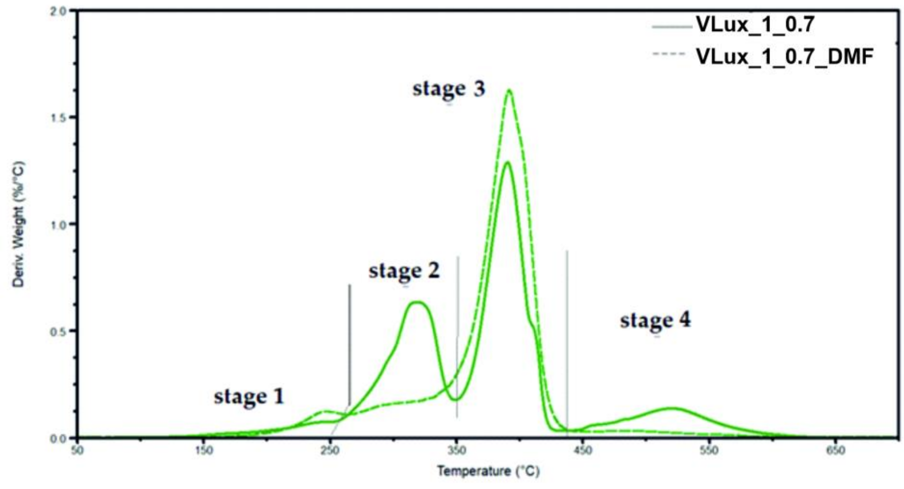

(**a**)

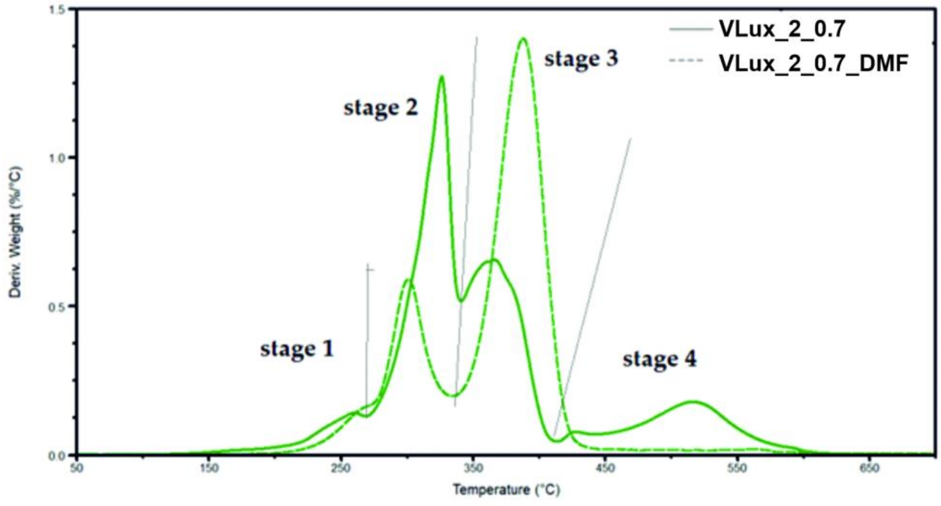

(**b**)

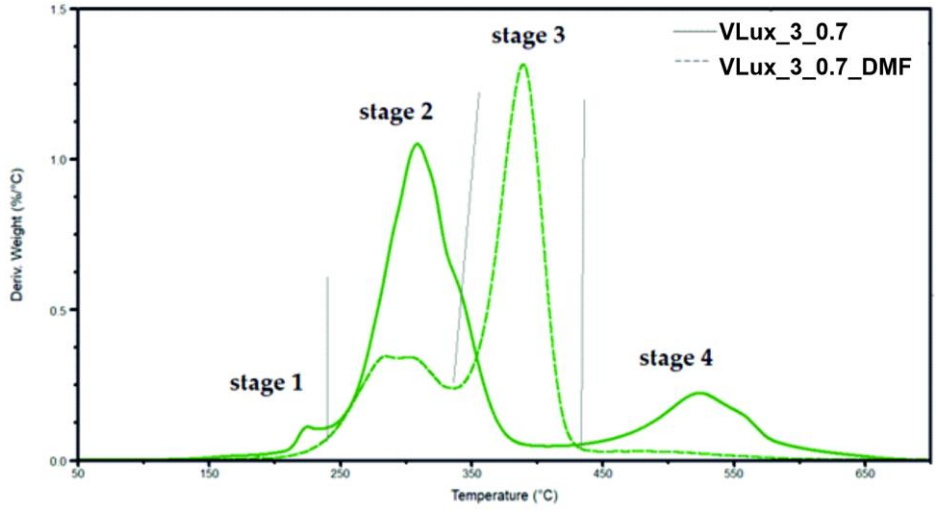

(**c**)

Figure S4. DTG curves of foams before and after exposure to DMF: (**a**) VLux_1_0.7, (**b**) VLux_2_0.7 and (**c**) VLux_3_0.7.
